# Anti-Obesity Effect of Hot Water Extract of Barley Sprout through the Inhibition of Adipocyte Differentiation and Growth

**DOI:** 10.3390/metabo11090610

**Published:** 2021-09-08

**Authors:** Myeong-Jin Kim, Hye-Won Kawk, Sang-Hyeon Kim, Hyo-Jae Lee, Ji-Won Seo, Jong-Tae Kim, Seung-Hee Jang, Min-Jeong Kim, Young-Min Kim

**Affiliations:** 1 Department of Biological Science and Biotechnology, College of Life Science and Nano Technology, Hannam University, Deajoen 34054, Korea; jiny2415@naver.com (M.-J.K.); gyp03416@daum.net (H.-W.K.); ssho26@naver.com (S.-H.K.); canopus310@naver.com (H.-J.L.); ddongji28@naver.com (J.-W.S.); 2 Teazen Co., Ltd., Haenam 59017, Korea; tea2000@teazen.co.kr (J.-T.K.); hqtop@nate.com (S.-H.J.); miri2805@naver.com (M.-J.K.)

**Keywords:** anti-obesity, barley sprout, saponarin, fatty liver, glucose uptake

## Abstract

Barley sprouts are known to have several effective physiological activities. In this study, the anti-obesity effect of a barley sprout hot water extract (BSE) was confirmed. Saponarin was quantitatively analyzed in BSE using HPLC, and the inhibitory effect on 3T3-L1 pre-adipocyte differentiation into adipocytes was confirmed by Oil Red O staining, TG assay, and Western blotting. In addition, the inhibitory effect of BSE on adipocyte growth was confirmed through glucose uptake and lipolysis of adipocytes. C57/BL/6N mice were induced to obesity with a high-fat diet, and BSE was administered to confirm the effect on an animal model. Weight gain, morphological changes in adipose tissue, changes in the food efficiency ratio, and blood biochemical changes were observed, and an improvement effect on fatty liver was confirmed. As a result, the anti-obesity effect of BSE was confirmed in vitro, and it was confirmed that this effect was also effective in vivo and that it could be helpful in the treatment of obesity-related diseases.

## 1. Introduction

The prevalence of obesity is increasing worldwide. From 1980 to 2015, the obese population increased by 80%, and this rapid increase in obesity is threatening human health beyond the problems of one’s appearance [1]. Obesity is known to cause high blood pressure, fatty liver, and various diseases of the digestive system [2,3]. In addition, excessive accumulation of adipose tissue leads to insulin resistance, which leads to type 2 diabetes, suggesting that obese people are at risk of complications from diabetes [4]. Obesity has even been found to be correlated with an increased risk of cancer [5,6,7]. Additionally, obesity itself causes stress, and it is known that stress causes eating disorders, sleep deprivation, and endocrine system abnormalities, which can also become further causes of obesity [8]. Therefore, obesity needs to be controlled to improve individual quality of life, reduce social costs, and form a healthy society.

There are many ways to treat obesity. Essentially, there are ways to reduce the amount of food eaten and increase the amount of activity undertaken, but this involves a lot of patience and pain [9]. In addition, intake of refined carbohydrates is also known as one of the causes of obesity so avoiding refined carbohydrates is one way to treat obesity. It can also be controlled through healthy functional foods and drugs [10,11]. Controlling obesity through drugs and healthy functional foods is known to be effective [12,13,14]. However, since they control obesity through various metabolic reactions and are accompanied by various side effects, people who want to control obesity through drugs and healthy functional foods are required to select an obesity control agent that is suitable for them, and broader research on anti-obesity strategies regarding anti-obesity drugs and healthy functional foods is needed for the establishment of obesity treatment [15,16,17,18,19].

In this respect, barley sprouts are noteworthy for their role in healthy functional foods or drugs used for obesity control. Many plant materials are known to help with weight control, and sprouted plants are also known to help with weight control [20,21,22]. Sprouted plants are known to have various physiologically active functions because nutrients used for growth after germination are densely concentrated in the plant [23]. Barley sprouts are known to be effective in alcoholic fatty liver, blood cholesterol, and immune enhancement and contain various polyphenols; many polyphenols are known to be effective in preventing cardiovascular diseases [24,25,26,27,28,29]. In addition, barley sprouts contain various flavonoids, and, among them, saponarin from barley sprouts has been attracting attention. Saponarin is known to have anti-diabetic, antioxidant, anti-inflammatory, and anti-bacterial effects; when it was used to treat 3T3-L1 pre-adipocytes, lipid synthesis was reduced, suggesting an effect on obesity [30,31,32,33,34,35].

Obesity is controlled by various metabolic activities in the body. The growth of adipose tissue depends on the differentiation and growth into adipocytes [36]. C/EBPα and PAPRγ are known as transcription factors that express adipocyte-differentiation-inducing proteins, and differentiated cells initiate lipid synthesis by activating ACC and FAS through decreased APMK activity [37,38,39,40]. Additionally, adipocytes and muscle cells are stimulated by insulin to control blood sugar [41]. When insulin stimulation occurs, lipolysis of adipocytes is inhibited, glucose uptake is induced, and lipid synthesis is promoted, which causes adipocytes to enlarge [42,43,44,45]. Therefore, inhibition of the differentiation of pre-adipocytes, suppression of the supply of glucose required for lipid synthesis in adipocytes, and activation of lipolysis can help control obesity.

In this study, basic research was conducted on the development of anti-obesity healthy functional foods and drugs using barley sprout. Barley sprouts were extracted under hot-water conditions, and the anti-obesity mechanism was confirmed in vitro using 3T3-L1 pre-adipocytes; the anti-obesity effect was confirmed in vivo through a C57/BL/6N mouse obesity model.

## 2. Results

### 2.1. Quantitative Analysis of Saponarin in BSE

**Figure 1:**
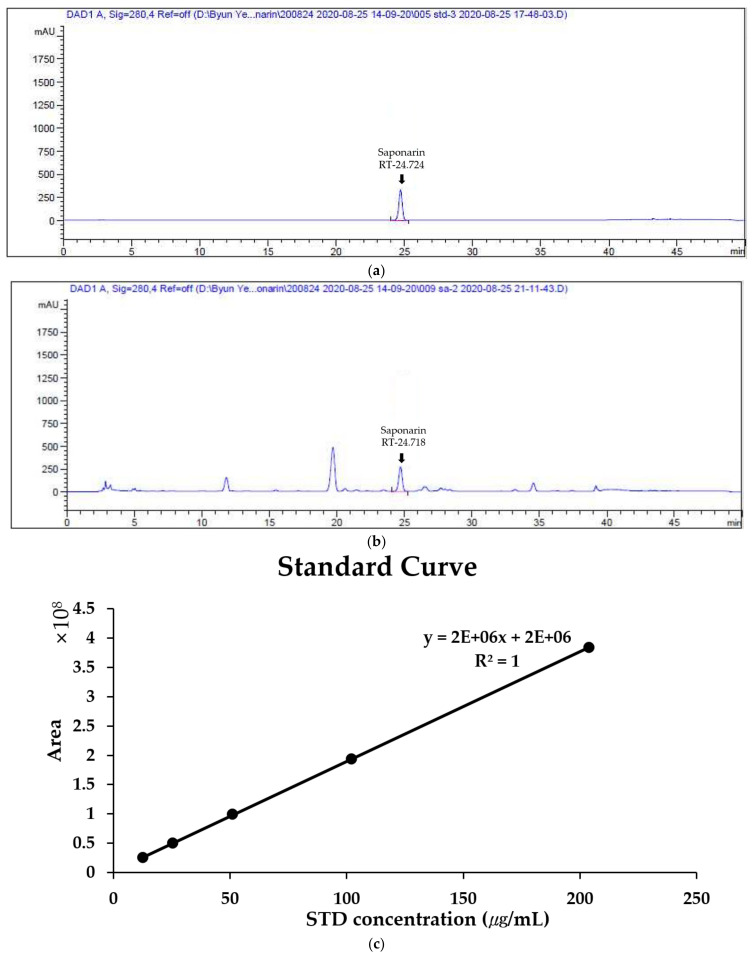
Quantitative analysis of saponarin in hot-water barley sprout extract (BSE) by HPLC (at 280 nm). (**a**) HPLC profile of saponarin (250 μg/mL). (**b**) HPLC profile of BSE (10 mg/mL). (**c**) Standard curve of saponarin at 12.75, 25.5, 51, 102, and 204 μg/mL.

As a result of analyzing the saponarin standards and BSE at 280 nm using HPLC, a peak was confirmed at the same retention time as that of saponarin standard solution and BSE. (Figure 1a,b) This confirmed that saponarin was contained in BSE. By calculating a standard curve through the peak areas of saponarin at 12.75, 25.5, 51, 102, and 204 μg/mL using HPLC, it was confirmed that the saponarin in BSE was at a concentration of 4.79 mg/g (Figure 1c).

### 2.2. Inhibitory Effect of BSE Differentiation into Adipocytes without Cytotoxicity

**Figure 2:**
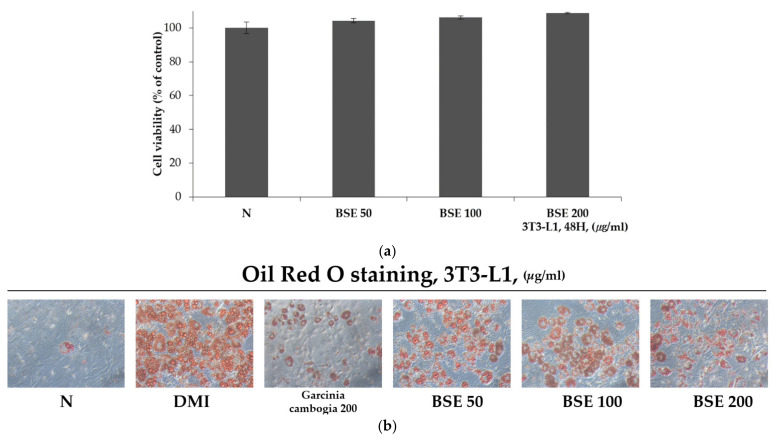

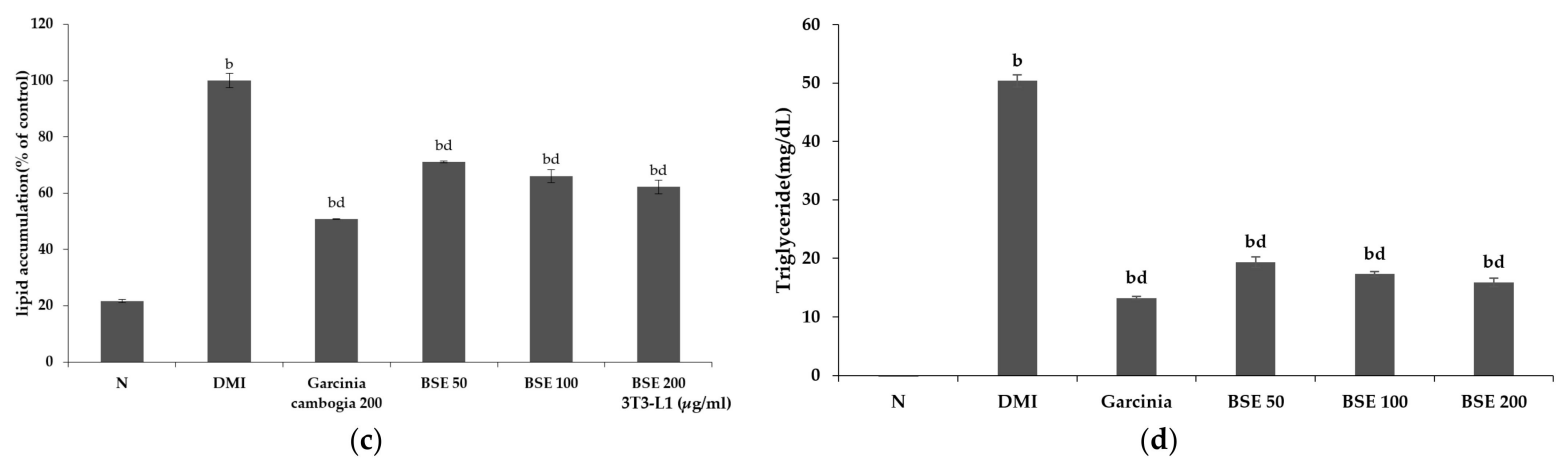
Differentiation inhibitory effect of BSE on 3T3-L1 cells. (**a**) Cytotoxicity through WST-1 assay on 3T3-L1 cells of BSE. (**b**) Morphological observation of lipid droplets in adipocytes through Oil Red O staining (100×, Axiovert 100, Oberkochen, Germany). (**c**) Oil Red O staining levels obtained by measuring absorbance at 500 nm with 100% isopropanol. (**d**) Measurement of intracellular triglyceride content. Statistical analysis was performed using a *t*-test. ^b^ *p* < 0.01 compared with Group N. ^d^ *p* < 0.01 compared with the DMI group. N: no substance treatment; DMI: 1 µM dexamethasone, 0.5 mM 3-isobutyl-1-methylxanthine, and 1 μg/mL of insulin; Gar: DMI + Garcinia cambogia; BSE: DMI + barley sprout hot-water extract.

In order to check the cytotoxicity of BSE to 3T3-L1 cells, each concentration of BSE (50, 100, and 200 μg/mL) was prepared and incubated with the cells for 48 h, and the cells were then treated with water-soluble tetrazolium-1 (WST-1). After 2 h, the absorbance of formazan, produced using WST-1, was observed at a wavelength of 450 nm to confirm cell viability. As a result, it was confirmed that the cell viability of 3T3-L1 cells was over 95% in all the groups treated with different concentrations of BSE, and it was confirmed that BSE was not toxic to the 3T3-L1 cells (Figure 2a). In addition, in order to confirm the inhibitory effect of BSE differentiation on adipocytes, 3T3-L1 pre-adipocytes were treated with DMI (a cocktail of dexamethasone, 3-isobutyl-1-methylxanthine, and insulin) to induce differentiation and treated with BSE and Garcinia cambogia extract, which is known to have anti-obesity effects [46]. Group N did not induce differentiation and was not treated with any substance. After differentiation for 8 days, the differentiated cells were identified through Oil Red O staining. Oil Red O stains hydrophobic substances in red and stains lipids synthesized in droplet form from differentiated adipocytes. As a result of the experiment, it was confirmed that Oil Red O staining stained the BSE-treated group less than the DMI group at all concentrations (Figure 2b). The stained Oil Red O solution was dissolved in 100% isopropanol, and the staining intensity was measured with a wavelength of 500 nm; it was confirmed that the Oil Red O staining of the group treated with 200 μg/mL of BSE, compared to DMI, was 61% (Figure 2c). In addition, a TG assay was performed to measure the triglyceride synthesized in differentiated adipocytes. As a result of the experiment, the triglyceride was measured to be lower in all the substance-treated groups than in DMI, and 15 mg/dl of triglyceride was measured at a concentration of 200 μg/mL in BSE. It was confirmed that the triglyceride synthesis was reduced to a level of 31% of DMI (Figure 2d). In Group N, in which we did not induce differentiation, Oil Red O staining and triglyceride synthesis were significantly reduced compared to all the other differentiation induction groups. Through these results, it was confirmed that BSE had the effect of inhibiting differentiation into adipocytes or inhibiting lipid synthesis.

### 2.3. Effect of BSE on Adipocyte-Differentiation-Related Protein Expression Change

**Figure 3:**
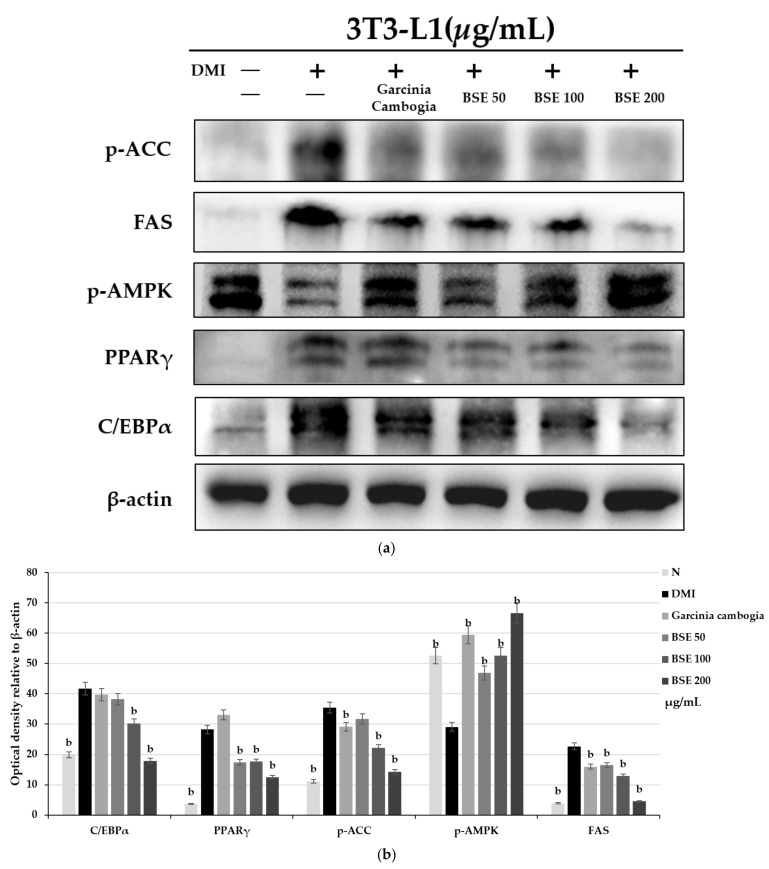
Changes in 3T3-L1 intracellular protein expression caused by hot-water barley sprout extract (BSE). (**a**) Western blotting and (**b**) semi-quantitative analysis. Statistical analysis was performed using a *t*-test. ^b^ *p* < 0.01 compared with group DMI. p-ACC, phospho-acetyl-CoA carboxylase; FAS, fatty acid synthase; p-AMPK, phospho-AMP-activated protein kinase; PPARγ, peroxisome proliferator-activated receptor gamma; C/EBPα, CCAAT/enhancer-binding protein alpha.

To confirm the expression of proteins involved in the differentiation of adipocytes, Western blotting was performed by extracting cultured proteins through treatment with DMI, BSE (50, 100, and 200 μg/mL), and garcinia (200 μg/mL) in parallel. C/EBPα and PPARγ are important transcription factors in the differentiation into adipocytes. As their expression increases, proteins necessary for differentiation are generated and the differentiation proceeds. AMPK is a kinase involved in energy metabolism; its activity decreases in anabolic processes such as lipid synthesis, and it increases the expression of ACC and FAS required for lipid synthesis. As a result of the experiment, it was confirmed that the expression levels of C/EBPα and PPARγ were decreased during BSE treatment, while the expression of p-AMPK, an activated form of AMPK, was increased compared to DMI during the BSE treatment. Accordingly, the expression of p-ACC and FAS also showed a tendency to decrease when the BSE treatment was compared to DMI (Figure 3). As the downregulation of proteins involved in adipocyte differentiation was observed, it was confirmed that BSE had an inhibitory effect on adipocyte differentiation.

### 2.4. Inhibitory Effect of BSE on Glucose Uptake in Adipocytes

**Figure 4:**
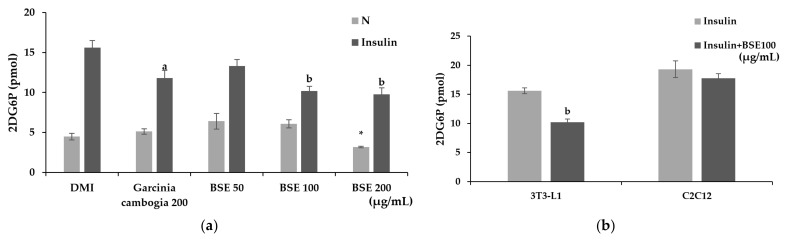
Changes in glucose uptake caused by hot-water barley sprout extract (BSE). (**a**) Changes in the glucose uptake of BSE with and without insulin stimulation in 3T3-L1 cells. (**b**) Comparison of changes in glucose uptake following insulin stimulation by 3T3-L1 and C2C12 cells. Statistical analysis was performed using one-way and two-way analysis of variance (ANOVA). * *p* < 0.05 compared with DMI group. ^a^ *p* < 0.05 and ^b^ *p* < 0.01 compared with the DMI + Insulin group. N: no substance treatment; DMI: 1 µM dexamethasone, 0.5 mM 3-isobutyl-1-methylxanthine, and 1 μg/mL of insulin; G: Garcinia cambogia at 200 μg/mL.

Glucose uptake was confirmed by measuring the uptake of 2-deoxyglucose (2-DG), which is transported like glucose but, unlike glucose, is not used for metabolism. To confirm the effect of BSE on inhibiting glucose uptake, differentiated adipocytes were treated with BSE and Garcinia cambogia while activating glucose uptake with insulin. As a result of the experiment, no significant change in glucose uptake was confirmed when treated with Garcinia cambogia or BSE at 50 or 100 μg/mL compared to Group N, but a significant decrease was observed at the concentration of BSE 200 μg/mL. However, when the glucose uptake was stimulated by insulin, a significant decrease in the glucose uptake was measured in all the substance-treated groups compared to the insulin-only treatment group. The BSE 50 μg/mL treatment showed lower glucose uptake reduction than that of Garcinia cambogia, but at BSE concentrations above 100 μg/mL, it showed a higher glucose uptake reduction than the Garcinia cambogia treatment did (Figure 4a). Additionally, the glucose uptake of C2C12-differentiated myotubes and 3T3-L1-differentiated adipocytes was confirmed. The adipocytes showed that the glucose uptake was reduced when BSE was treated as in the previous experiment, reducing it to 65% of that of the insulin-treated group. However, in the case of the myotube, there was no significant difference between the group treated with only insulin and the group treated with BSE (Figure 4b). As a result, it was confirmed that BSE reduced insulin-stimulated glucose uptake in an adipocyte-specific manner.

### 2.5. Confirmation of Lipolysis Effect by BSE

**Figure 5:**
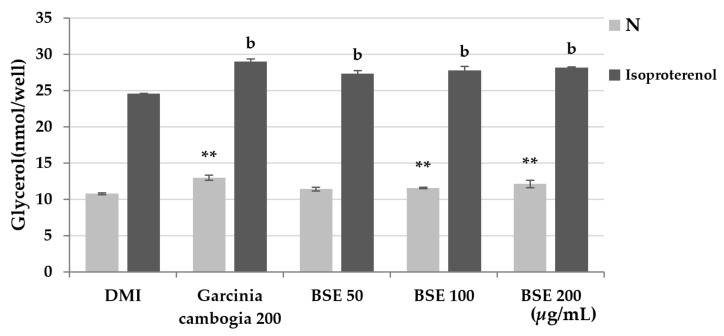
Effect of lipolysis induction by the hot-water extract of barley sprout (BSE). Statistical analysis was performed using one-way and two-way analysis of variance (ANOVA). ** *p* < 0.01 compared with DMI group. ^b^ *p* < 0.01 compared with DMI + isoproterenol group. N: no substance treatment; DMI: 1 µM dexamethasone, 0.5 mM 3-isobutyl-1-methylxanthine, and 1 μg/mL of insulin.

Lipolysis mainly occurs in adipocytes. Lipolysis in adipocytes can inhibit the hypertrophy of adipocytes by preventing excessive lipid accumulation in cells. During lipolysis, triglycerides are decomposed into free fatty acids and glycerol and released into the blood or medium. Lipolysis can be measured by measuring the glycerol released into the medium at 570 nm using a probe. Lipolysis is a very slow process. In order to minimize the exposure of adipocytes to the experimental buffer and to quickly confirm the effect of the substance, the experiment was conducted by promoting lipolysis with isoproterenol. To confirm the lipolysis effect, differentiated 3T3-L1 cells were treated with BSE and Garcinia cambogia, and isoproterenol was added. As a result of the experiment, it was confirmed that lipolysis occurred 14% more in BSE than in the DMI group, and lipolysis increased by 17% when treated with Garcinia cambogia (Figure 5). Through this, the effect of BSE inducing the lipolysis of adipocytes was confirmed.

### 2.6. Confirmation of Changes in Body and Tissue Weight and Food Intake of Experimental Animals by BSE

In order to confirm the anti-obesity effect in the animal experimental model of BSE, C57/BL/6N mice were administered a high-fat diet for each concentration of BSE (25, 50, and 100 mg/kg/day), and Garcinia cambogia (100 mg/kg/day) was administered orally; changes were confirmed after 8 weeks. As a result, significant weight gain was observed in all obesity induction groups using a high-fat diet compared to NFD, and a dose-dependent decrease in weight gain was confirmed in the BSE-treated group (25, 50, and 100 groups) compared to the HFD group. In particular, the weight gain was reduced by 32% compared to HFD in the 100 group. Food intake also decreased in the BSE-administered group (25, 50, and 100 groups) and the Gar group, while a significant decrease in the food efficiency ratio (FER) based on weight gain and food intake was confirmed in the 100 group. The weight of the liver was significantly reduced in the 50, 100, and Gar groups compared to the HFD group. In addition, a significant reduction in the weight of all adipose tissue was observed in the 100 group compared to the HFD group (Table 1). Thus, it was confirmed that BSE decreased the FER of the experimental animals and decreased weight gain due to obesity.

### 2.7. Confirmation of Blood Biochemical Changes by BSE

The serum was separated from the blood collected by sacrificing experimental animals that had been tested for 8 weeks, and blood biochemical tests were performed. ALT and AST indicate liver inflammation levels. These are high in fatty livers, which can be induced by obesity. As a result of the experiment, the ALT and AST levels showed a dose-dependent decreasing trend in the BSE-treated group (25, 50, and 100 mg/kg/day) when compared with the HFD group, and a significant decrease trend was observed in the 100 group. In addition, the AST level of the 100 group was so low that it was insignificant compared with the NFD group. In addition, the decrease in ALT and AST was also confirmed in the Garciana-cambogia-administered group. Garcinia cambogia has been reported to have hepatotoxicity, for example, causing acute hepatitis, and it is known that the administration of the clinically recommended dose also shows toxicity to the liver in experimental animals [20,47,48,49]. In this study, it is worth noting that the experiment was conducted by administering a much lower dose of Garcinia cambogia than the dose considering the body surface area. As for the glucose level, an increase in blood glucose was observed in the HFD and Gar groups compared to the NFD group. However, the BSE-treated group showed an insignificant low level compared to the NFD group, and the glucose level of the BSE-treated group showed a significant decrease in blood glucose when compared with the HFD group. The concentration of triglyceride in the blood was not increased compared to the NFD group in all the groups except the HFD group, and triglyceride was decreased in the Gar and 100 groups compared to the HFD group. The total cholesterol level was increased compared to the NFD group in all concentration groups, but total cholesterol decreased in the 100 group compared to the HFD group. HDL was confirmed to be elevated in all concentration groups compared to the NFD group, and no significant change was observed between the groups that were fed a high-fat diet. An increase in the LDL level compared to that of the NFD group was confirmed in all obesity-induction groups, and a decrease was confirmed only in the Gar group compared to the HFD group. The atherogenic index (AI) is a number related to cardiovascular disease based on the HDL ratio of total cholesterol [50]. As a result of the experiment, an increase in the AI level was confirmed in all obesity-induction groups compared to the NFD group, and an overall decrease in the AI level was confirmed, although it was not significant in the Gar- and BSE-treated groups compared to the HFD group (Table 2). Therefore, BSE was confirmed to have the effect of improving the blood’s biochemical levels related to obesity, and it was confirmed that the improvement tendency appeared even when there was an insignificant difference.

### 2.8. Confirmation of Changes in Leptin and Adiponectin Levels by BSE

**Figure 6:**
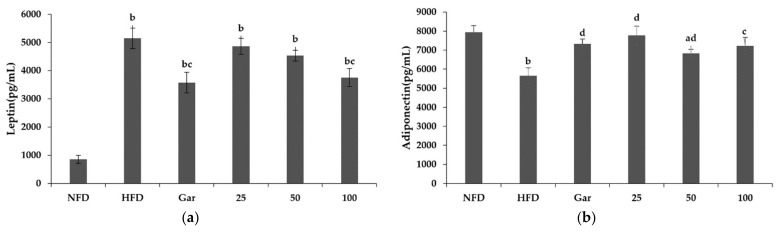
Changes in the leptin and adiponectin levels caused by hot-water barley sprout extract (BSE). (**a**) Leptin levels in serum. (**b**) Adiponectin levels in serum. Statistical analysis was performed using a *t*-test. ^a^ *p* < 0.05 and ^b^ *p* < 0.01 compared with the NFD group. ^c^ *p* < 0.05 and ^d^ *p* < 0.01 compared with the HFD group. NFD: normal-fat diet; HFD: high-fat diet; Gar: Garcinia cambogia 100 mg/kg/day; 25, BSE 25 mg/kg/day; 50, BSE 50 mg/kg/day; 100, BSE 100 mg/kg/day. Values are the mean ± SD of six mice per group.

The leptin and adiponectin levels were confirmed through the serum of the experimental animals. Leptin is a hormone that regulates satiety and metabolism, secreted by fat cells, and its level is known to increase when obese. As a result of the experiment, it was confirmed that leptin was increased in all obesity-induction groups compared to the NFD group, and leptin levels were decreased in the Gar- and BSE-treated groups compared to the HFD group. We measured a leptin level of 3578 pg/mL in the Gar group and 3756 pg/mL in the 100 group, which showed a decrease of 31% (Gar) and 27% (100) compared to that of the HFD group (5145 pg/mL) (Figure 6a). Adiponectin, along with leptin, is a major biomarker identified in obesity studies, and it is known to regulate appetite and help with insulin resistance. It is known that the amount of adiponectin in the blood decreases during obesity, and the level of adiponectin in the blood increases when weight loss occurs. As a result of the experiment, there was an insignificant decrease in all the material treatment groups except for the 50 group compared to the NFD group; an increase in adiponectin was confirmed in the Gar- and BSE-treated groups compared to the HFD group (Figure 6b).

### 2.9. Effect of BSE on Adipose Tissue Histology and Adipocyte-Differentiation-Related Factors

**Figure 7:**
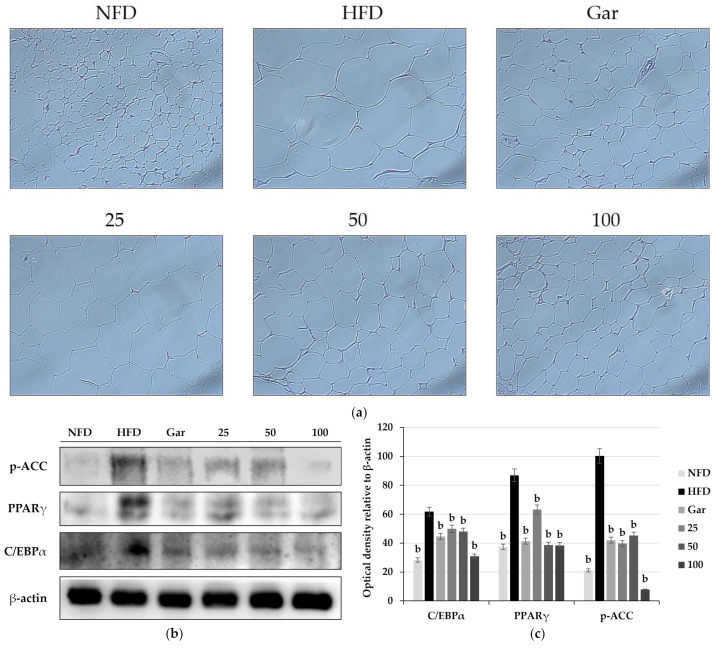
(**a**) Observation of changes in epididymal adipose tissue by H&E staining (400×, Axiovert100, Oberkochen, Germany). Changes in the expression of p-ACC, PPARγ, and C/EBPα in epididymal adipose tissue. (**b**) Western blotting and (**c**) semi-quantitative analysis. Statistical analysis was performed using a *t*-test. ^b^ *p* < 0.01 compared with the HFD group. NFD: normal-fat diet; HFD: high-fat diet; Gar: Garcinia cambogia 100 mg/kg/day; 25: hot-water barley sprout extract (BSE) 25 mg/kg/day; 50: BSE 50 mg/kg/day; 100: BSE 100 mg/kg/day.

The experiments were conducted using epididymal fat collected by sacrificing experimental animals. Morphological changes were confirmed by the H&E staining of the epididymal fat of experimental animals. As a result of the experiment, it was observed that a large amount of lipids was accumulated in adipocytes in the obesity-induction group compared to the NFD group, resulting in enlarged adipocytes. However, compared to the HFD group, the hypertrophy of adipocytes was significantly reduced in the Gar group, and it was confirmed that the size of adipocytes decreased in a dose-dependent manner in the BSE-treated group (25, 50, and 100 groups) (Figure 7a). In addition, as a result of Western blotting using adipose fat, it was confirmed that the expression levels of p-ACC, C/EBPα, and PAPRγ were decreased in the Gar- and BSE-treated groups compared to the HFD group (Figure 7b).

### 2.10. Effect of BSE on Liver Histology and Adipocyte-Differentiation-Related Factors

**Figure 8:**
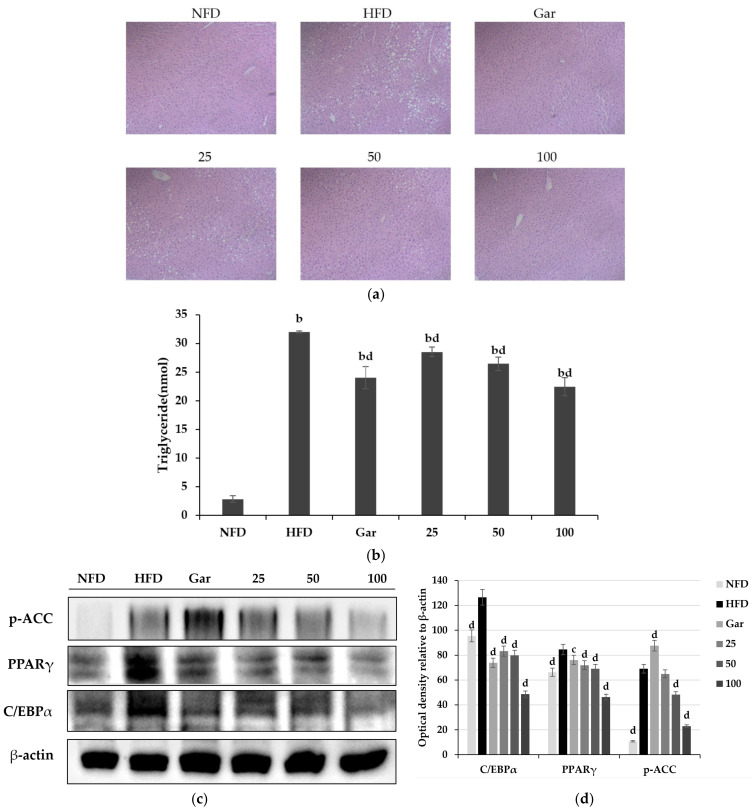
Improvement effect of hot-water barley sprout extract (BSE) on fatty liver. (**a**) Histological changes in liver tissue through H&E staining (400×, Axiovert100, Oberkochen, Germany). (**b**) Measurement of triglyceride content in liver tissue. Changes in p-ACC, PPARγ, and C/EBPα expression in liver tissue. Values are the mean ± SD of six mice per group. (**c**) Western blotting and (**d**) semi-quantitative analysis. Statistical analysis was performed using a *t*-test. ^b^ *p* < 0.01 compared with NFD group. ^c^ *p* < 0.05 and ^d^ *p* < 0.01 compared with HFD group; NFD: normal-fat diet; HFD: high-fat diet; Gar: Garcinia cambogia at 100 mg/kg/day; 25: BSE 25 mg/kg/day; 50: BSE 50 mg/kg/day; 100: BSE 100 mg/kg/day.

The experiment was carried out using the livers of experimental animals tested for 8 weeks. H&E staining was performed on the collected livers for morphological observation. As a result of the experiment, hepatic lipid accumulation was not confirmed in the NFD group, but hepatic lipid accumulation was confirmed in all high-fat diet groups. However, it was observed that lipid accumulation was reduced in the Gar- and BSE-treated groups compared to the HFD group (Figure 8a). As a result of measuring the amount of lipid accumulation in the liver by TG assay, triglyceride reduction was confirmed in the Gar- and BSE-treated groups compared to the HFD group, and it was confirmed that the 100 group had a 70% reduction compared to the level of the HFD group (Figure 8b). In addition, Western blotting was performed to confirm the expression of proteins involved in the differentiation of the liver into fatty liver. As a result of the experiment, it was confirmed that the expression of C/EBPα and PAPRγ was decreased in the Gar- and BSE-treated groups compared to the HFD group (Figure 8c).

## 3. Discussion

Weight control drugs or functional foods are known to control obesity through various mechanisms. Obesity can be suppressed by mechanisms such as the inhibition of sugar absorption, the inhibition of fat absorption, appetite suppression, and adipocyte differentiation inhibition [51,52]. Therefore, the search for various anti-obesity substances and the analysis of anti-obesity mechanisms will enable people to more actively select anti-obesity strategies. This study is a basic study of hot-water barley sprout extract (BSE) as a healthy functional food and medicine to help people establish an anti-obesity strategy suitable for them.

Saponarin is a multifunctional flavonoid that is mainly found in barley sprouts, and it is known to have anti-obesity effects. Therefore, the content of saponarin in BSE was measured, and it was confirmed that it contained 4.79 mg/g. However, in a previous study, when barley sprouts were extracted with 70% EtOH, 11–14 mg/g of saponarin was extracted, so it can be seen that hot-water extraction conditions are not optimal for saponarin extraction [24]. However, the results were different for the anti-obesity effect. This study confirmed that BSE showed a stronger anti-obesity effect at a lower concentration than the 70% EtOH extract of previous studies [35]. Natural products show a difference in physiological activities, such as anti-obesity and anti-cancer effects, depending on the extraction conditions [53,54,55]. Through this study, it was confirmed that hot-water extraction was the optimal extraction condition for the development of healthy functional foods and drugs for the anti-obesity effect of barley sprouts. Additionally, BSE that contained less saponarin than the 70% EtOH barley sprout extract had a greater anti-obesity effect, suggesting that saponarin is not a major indicator of the anti-obesity effect of BSE. Although saponarin was not shown to be a major indicator of the anti-obesity effect of BSE, it was possible to estimate the various physiological effects of saponarin from BSE through qualitative analysis. In addition, this discussion indicates that the value of follow-up studies on substances with strong anti-obesity effects in BSE is high.

The anti-obesity effect of BSE was confirmed by an Oil Red O staining and TG assay, and it was confirmed that BSE showed a strong anti-obesity effect even at a low concentration. Additionally, it was confirmed by Western blotting that BSE reduced the expression of C/EBPα, PAPRγ, p-APMK, p-ACC, and FAS. Thus, it was confirmed that the anti-obesity effect of BSE was due to the inhibition of differentiation into adipocytes. In addition, the anti-obesity effect of BSE by inhibiting the growth of adipocytes was confirmed. It was confirmed that BSE activates lipolysis and decreases adipocyte-specific glucose uptake by insulin.

Insulin is necessary for blood sugar control, but it is known to help the growth of adipocytes [56]. However, it was confirmed that BSE suppressed the glucose supply required for lipid synthesis in adipocytes without interfering with normal blood glucose control of muscle cells through insulin. This suggests that it will be effective for obesity caused by overeating and high carbohydrate intake [57,58,59,60]. It was also confirmed that BSE activates lipolysis. Thus, it was confirmed that BSE has an anti-obesity effect by inhibiting adipocyte differentiation as well as adipocyte growth.

The anti-obesity effect of BSE was also confirmed in vivo. Upon administration of BSE, a decrease in weight gain and a decrease in FER (food efficiency ratio) were observed in a dose-dependent manner. FER is the ratio of increasing weight to food intake, and a decrease in FER means less weight gain compared to food intake [61,62,63,64]. As adipose tissue weight reduction, adipocyte growth inhibition through morphology, and differentiation inhibition through Western blotting were observed, it was confirmed that the anti-obesity effect of BSE was a factor in reducing FER.

In addition, it was confirmed that the anti-obesity effect of BSE can help to improve obesity-related metabolic diseases. The BSE-administered group showed improvements in glucose and triglyceride levels compared to the HFD group. In addition, the overall cholesterol level was reduced, and BSE seemed to help improve the AI (atherogenic index) level, which is closely related to cardiovascular diseases such as atherosclerosis [50]. Although no significant change in AI due to BSE was observed in this study, a dose-dependent decrease was observed, suggesting that high-dose BSE intake could help cardiovascular disease. In addition, as the improvement of leptin and adiponectin levels was confirmed when BSE was administered, BSE seems to be helpful in obesity-induced diabetes and dietary disorders [65,66,67,68]. It was also confirmed that BSE can help improve fatty liver due to obesity by reducing ALT and AST, which can reduce fatty liver due to obesity by inhibiting differentiation into fatty liver and reducing lipid accumulation [69].

In conclusion, through this experiment, it was confirmed that there are substances with high anti-obesity effects other than saponarin in BSE; BSE inhibited adipocyte differentiation and adipocyte growth and was effective in improving obesity-related metabolic diseases. Thus, the potential of BSE as a healthy functional food for obesity control was confirmed, and it is expected that it will be possible to develop BSE into a drug through the single isolation, identification, and standardization of the substances in BSE with major anti-obesity effects.

## 4. Materials and Methods

### 4.1. Reagent

This experiment was carried out by selecting Albarley seeds. The barley sprouts were cultivated by Teazen (Haenam, Korea) and used in the experiment. Water 20 times the weight of barley grown to 25 cm was added, extracted at 40 °C for 6 h, dried, and concentrated to 15–10 brix. After that, dextrin was mixed with concentrated barley sprout extract in a ratio of 6:4 at 80 °C and dried to obtain a hot-water extract powder of barley sprouts, which was stored at −20 °C. The hot-water extract of barley sprouts was dissolved in DMSO for each concentration (50, 100, and 200 μg/mL) and used in the experiment.

### 4.2. Quantitative Analysis of Saponarin in BSE Using HPLC

The mobile phases used for the HPLC analysis were: mobile phase A, MeOH (HPLC grade, Burdick & Jackson, Muskegon, US); mobile phase B, water (with 0.1% trifluoroacetic acid, Sigma-Aldrich, St. Louis, US). Saponarin standard solution was obtained by diluting saponarin (Cat#89794, PhytoLab, Vestenbergsgreuth, Germany) in 80% MeOH, and BSE was also diluted in 80% MeOH at a concentration of 10 mg/mL. HPLC analysis was performed using an Agilent 1260 Infinity series instrument (Agilent, Santa Clara, US) and Inno C18 (250 mm × 4.6 mm, 5 µm, YoungJin Biochrom, Seongnam-si, Korea). The ratio of the mobile phase used in the experiment is shown in Table 3, and the absorbance was measured at a wavelength of 280 nm.

### 4.3. Cell Culture

3T3-L1 (mouse pre-adipocytes) and C2C12 (mouse myoblast) cells were obtained from American Type Culture Collection (ATCC, Washington, USA). 3T3-L1 cells were cultured using DMEM (Dulbecco’s modified Eagle medium) containing 10% bovine calf serum (HyClone Laboratories Inc, Marlborough, UK) and 1% antibiotic, and C2C12 cells were cultured using DMEM containing 10% fetal bovine serum (HyClone Laboratories Inc., Marlborough, UK) and 1% antibiotic. Cells were cultured in an incubator under the conditions of 37 °C and 5% CO2, and 3T3-L1 was suspended through trypsin/EDTA every 72 h, seeded at a concentration of 3 × 10^4^ cells/mL, and subcultured. C2C12 was seeded at a concentration of 5 × 10^4^ cells/mL and subcultured.

### 4.4. WST-1 Assay

3T3-L1 cells were seeded in a 12-well plate at a concentration of 1 × 10^5^ cells/mL. After incubation for 24 h, the hot-water extract of barley sprouts was treated at each concentration (50, 100, and 200 μg/mL). After 48 h of BSE treatment, 10% of the medium volume of water-soluble tetrazolium-1 (WST-1) solution was added to each well. After that, incubation was performed for 2 h to generate formazan. Then, after transferring 100 μL of the medium to a 96-well plate, the absorbance of formazan was measured at a wavelength of 450 nm to observe cell viability.

### 4.5. Differentiation Induction

We incubated 3T3-L1 cells in DMEM containing 10% BCS until confluent. Then, 0.5 mM of 3-isobutyl-1-methylxanthine, 1 µM of dexamethasone, and 1 μg/mL of insulin (DMI solution) were added to induce differentiation. In addition, BSE (50, 100, and 200 μg/mL) and Garcinia cambogia (200 μg/mL) were concurrently treated. After 48 h of DMI treatment, we then used a DMEM medium containing 1 μg/mL insulin and 10% FBS until the differentiation had proceeded sufficiently and then treated the cells with BSE or Garcinia cambogia. Group N was not treated with DMI or any other substance.

### 4.6. Oil Red O Staining

3T3-L1 cells seeded in a 24-well plate at 1 × 10^5^/mL were treated with BSE (50, 100, and 200 μg/mL) and Garcinia cambogia (200 μg/mL), along with differentiation induction through DMI. After sufficiently differentiating the 3T3-L1 cells, they were fixed with 10% formalin, and 60% isopropanol was added. Then, after staining with Oil Red O solution for 10 min, they were washed 5 times with PBS and then observed morphologically. In addition, the staining of Oil Red O was dissolved using 100% isopropanol and observed with a wavelength of 500 nm.

### 4.7. TG Assay

Triglyceride content was measured using a triglyceride assay kit (ab65336, ABcam, Cambridge, UK). After incubating the 3T3-L1 cells seeded on a 6-well plate until confluence, DMI and BSE (50, 100, and 200 μg/mL) or Garcinia cambogia (200 μg/mL) were added to induce differentiation. After lysing the differentiated cells with 5% NP-40, the reaction was repeated at 90 ℃ for 5 min. In the case of tissue, 5% NP-40 was added and sonicated to dissolve the tissue. After that, the lysate was diluted 10 times, reacted with lipase, and then reacted with a probe for 60 min at RT in a light-excluding environment. Then, the amount of triglyceride was confirmed by measuring the absorbance at 570 nm.

### 4.8. Western Blotting

3T3-L1 was cultured in a state of confluence in a 6-well plate, and differentiation was induced by treatment with DMI, BSE, and Garcinia cambogia. The differentiated 3T3-L1 cells were lysed using RIPA buffer containing a protease inhibitor cocktail. The cell lysate was centrifuged at 14,000 rpm at 4 °C for 20 min to separate the supernatant. In the case of tissue, a RIPA buffer was added and sonicated to obtain lysate, then centrifuged to separate the supernatant. The isolated protein was quantified using the Bradford assay, denatured at 100 °C for 10 min, and stored at −75 °C. The SDS phase was identified using 8% and 10% acrylamide gels, then transferred to a nitrocellulose membrane. The transferred membrane was blocked with 4% BSA for 2 h, and the primary antibody of the protein to be confirmed was reacted overnight at 4 ℃ with PPARγ, C/EBPα (Santa Cruz Biotechnology, Dallas, Texas, US), fatty acid synthase (FAS), phospho-acetyl-CoA carboxylase (p-ACC), phospho-AMP-activated protein kinase (p-AMPK), and β-actin (Cell Signaling Technology, Danvers, US). Additionally, after reacting the secondary antibody for 2 h, protein expression was observed using a chemiluminescent substrate (SuperSignal™ West Pico PLUS Chemiluminescent Substrate, Thermo Fisher, Middlesex, MA, USA). Western blotting images were semi-quantified using ImageJ (National Institutes of Health, Bethesda, MD, USA).

### 4.9. Glucose Uptake Assay

3T3-L1 cells were cultured in a 96-well plate and treated with DMI to induce differentiation. C2C12 was differentiated with DMEM medium containing 2% horse serum until confluence after culturing in a 96-well plate. After the differentiation was complete, serum-free DMEM was added and left overnight to induce starvation. After that, Krebs-Ringer-Phosphate-HEPES Buffer (KRPH Buffer, iNtRON Biotechnology, Sungnam-si, Korea) containing 2% BSA was added and incubated for 40 min. After that, 1 µM of insulin, BSE, and Garcinia cambogia was added and incubated for 20 min. After this step, we treated it with 10 mM of 2-deoxyglucose (2-DG) and incubated it for 20 min. After washing with PBS, the experiment was performed using the Glucose Uptake Colorimetric Assay Kit (Cat. K676-100, BioVision, Milpitas, CA, USA).

### 4.10. Lipolysis Assay

In this experiment, the lipolysis level was measured using the BioVision Lipolysis (3T3-L1) Colorimetric Assay Kit (Cat. K577-100, BioVision, Milpitas, CA, USA). After seeding 3T3-L1 cells in a 96-well plate, DMI was added to induce differentiation. Additionally, 1.5 µL of 10 µM isoproterenol and BSE were added together to stimulate lipolysis for 3 h. Then, the probe was added and reacted for 30 min, and absorbance was measured at a wavelength of 570 nm to measure lipolysis.

### 4.11. Breeding and Diet of Experimental Animals

The experiments were conducted with 5-week-old male C57/BL/6N mice (ENVIGO, USA). The experimental animals were acclimatized to a new environment for 1 week, and the experiment was conducted by randomly dividing the animals into 6 groups of 6 animals each. During the experiment, the animals were allowed to freely ingest feed and water. Experiments were conducted in the 6 groups of NFD, HFD, Gar, 25, 50, and 100. The NFD group was fed a normal fat diet (10% fat kcal, from Purina, St. Louis, US). All the groups except for the NFD group were fed a high-fat diet (60% fat kcal, ENVIGO, Indianapolis, IN, USA). The feed composition is shown in Table 4. The HFD group was administered PBS with a high-fat diet, and the Gar group was administered Garcinia cambogia (100 mg/kg/day). The 25 group was administered BSE at 25 mg/kg/day, the 50 group was administered BSE at 50 mg/kg/day, and the 100 group was administered BSE at 100 mg/kg/day. Garcinia cambogia was diluted in water and BSE was diluted in PBS according to each concentration and administered. Experimental animals were kept in an environment of 21~25 °C, 45~55% humidity, with light and dark cycles every 12 h. PBS, Garcinia cambogia, and BSE were administered orally at the same time every day for 8 weeks. All the animal experiments were conducted with the approval of the Hannam University Animal Experimental Ethics Committee (2020-6 (HNU2020-6), Daejeon, Korea).

### 4.12. Body Weight and Food Efficiency Measurements

The weight of the experimental animals was measured once every week on the same day and at the same time. Feed was given daily. Feed intake was observed by measuring the weight of feed once a week and measuring the weight of the remaining feed after 24 h. The food efficiency ratio (FER) was calculated by dividing the weight gain by feed intake during the experiment.

### 4.13. Blood and Tissue Collection and Analysis

After 8 weeks of experimentation, blood and tissues were collected by the sacrificing experimental animals that had fasted for more than 12 h. The abdomen of the experimental animal was opened, and blood was collected through the abdominal vein. The collected blood was coagulated at RT for 30 min and then centrifuged at 14,000 RPM for 20 min to separate the serum. The concentrations of alanine aminotransferase (ALT), aspartate aminotransferase (AST), glucose, triglycerides, total cholesterol, low-density lipoprotein (LDL) cholesterol, and high-density lipoprotein (HDL) cholesterol in the collected serum were measured using a biochemical analyzer (AU480 Chemistry Analyzer, Beckman Coulter, Brea, CA, USA). Leptin was measured using the Leptin Mouse ELISA kit (Cat. ab199082. Abcam, Cambridge, UK), and adiponectin was measured using the Mouse Adiponectin ELISA kit (Cat. ab108785, Abcam, UK). After blood collection, the liver, abdominal fat, epididymal fat, visceral fat, and subcutaneous fat were collected and stored at −75 °C. Additionally, liver and epididymal fat were fixed in 10% formalin for more than 24 H. After dehydrating the tissue using EtOH (70%, 80%, 90%, and 100%) and xylene, the tissue was fixed using paraffin. After cutting the paraffin-fixed tissue to make a section with a thickness of 4 μm, hematoxylin and eosin (H&E) staining was performed.

### 4.14. Significance Analysis

All the experiments were repeated at least 3 times. The mean of the experimental results, the calculation of standard error, and significance evaluation using *t*-tests and ANOVA (analysis of variance) were performed using the SPSS 21.0 program (IBM-SPSS, Chicago, IL, USA). The significance between groups was divided into levels of *p* < 0.05 and *p* < 0.01.

## Figures and Tables

**Table 1:** Measurement of body and tissue weight, food intake, and FER.

Measurements	NFD ^1^	HFD ^2^	Gar ^3^	25 ^4^	50 ^5^	100 ^6^
Weight (g)						
1 week (start supplement)	22.86 ± 0.63	21.52 ± 0.29	21.48 ± 0.23	22.3 ± 0.74	22.17 ± 0.24	20.90 ± 0.45
9 weeks (end supplement)	26.77 ± 0.30	36.87 ± 1.71 ^b^	32.78 ± 1.09 ^b^	34.48 ± 0.95 ^b^	33.60 ± 0.59 ^b^	31.37 ± 0.42 ^bc^
Weight gain (g)	3.90 ± 0.72	15.35 ± 1.48 ^b^	11.30 ± 1.01 ^bc^	12.18 ± 1.07 ^b^	11.43 ± 0.72 ^bc^	10.47 ± 0.70 ^bc^
Intake						
Food intake (g/day)	2.57 ± 0.62	2.11 ± 0.47 ^b^	1.96 ± 0.7 ^b^	1.97 ± 0.44 ^bc^	1.88 ± 0.63 ^bc^	1.91 ± 0.91 ^b^
Energy intake (kcal/day)	10.3 ± 0.25	10.8 ± 0.24	10.01 ± 0.36	10.05 ± 0.22 ^c^	9.59 ± 0.32 ^c^	9.77 ± 0.46
FER ^7^	0.027 ± 0.002	0.129 ± 0.012 ^b^	0.102 ± 0.019 ^b^	0.110 ± 0.019 ^b^	0.108 ± 0.016 ^b^	0.097 ± 0.023 ^bc^
Tissue weight (g)						
Liver	1.10 ± 0.04	0.97 ± 0.05 ^b^	0.86 ± 0.02 ^d^	0.95 ± 0.04	0.91 ± 0.02 ^c^	0.88 ± 0.03 ^d^
Abdominal fat tissue	0.50 ± 0.03	1.94 ± 0.14 ^b^	1.72 ± 0.12 ^b^	1.85 ± 0.12 ^b^	1.57 ± 0.06 ^b^	1.40 ± 0.06 ^bc^
Epididymal fat tissue	0.11 ± 0.01 ^b^	0.37 ± 0.03 ^b^	0.31 ± 0.02 ^b^	0.28 ± 0.01 ^bc^	0.29 ± 0.02 ^b^	0.24 ± 0.01 ^bd^
Visceral fat tissue	0.71 ± 0.05 ^b^	1.88 ± 0.18 ^b^	1.42 ± 0.08 ^bc^	1.60 ± 0.11 ^b^	1.52 ± 0.05 ^b^	1.33 ± 0.04 ^bc^
Subcutaneous fat tissue	0.71 ± 0.07 ^b^	2.96 ± 0.36 ^b^	2.17 ± 0.15 ^b^	2.21 ± 0.24 ^b^	1.97 ± 0.08 ^bc^	1.58 ± 0.09 ^bd^

^1^ NFD: normal-fat diet. ^2^ HFD: high-fat diet. ^3^ Gar: Garcinia cambogia 100 mg/kg/day. ^4^ 25: BSE 25 mg/kg/day. ^5^ 50: BSE 50 mg/kg/day. ^6^ 100: BSE 100 mg/kg/day. ^7^ Food Efficiency Ratio (FER) = [weight gain (g/day)]/[food intake (g/day)]. The statistical analysis was carried out by the use of a *t*-test. ^b^ *p* < 0.01 compared with the NFD group. ^c^ *p* < 0.05 and ^d^ *p* < 0.01 compared with the HFD group. Values are the mean ± SD of six mice per group.

**Table 2:** Measurement of blood biochemical levels.

Measurements	NFD ^1^	HFD ^2^	Gar ^3^	25 ^4^	50 ^5^	100 ^6^
ALT (U/L)	38.75 ± 1.25	84.25 ± 3.54 ^b^	55 ± 3.11 b ^d^	75 ± 7.55 ^b^	68.5 ± 9.42 ^a^	57.5 ± 7.03 ^ac^
AST (U/L)	25 ± 0.58	73.5 ± 9.51 ^b^	32.5 ± 4.17 ^d^	49.5 ± 8.97 ^a^	56.75 ± 15.6	34.25 ± 6.3 ^c^
Glucose (mg/dL)	194 ± 13.68	304 ± 18.06 ^b^	259.5 ± 7.23 ^b^	223.5 ± 11.72 ^c^	244.75 ± 18.29	242 ± 14.16 ^c^
Total-Cholesterol (mg/dL)	85.5 ± 1.04	153.5 ± 6.22 ^b^	138.5 ± 4.91 ^b^	133.25 ± 9.72 ^b^	141 ± 3.76 ^b^	134.25 ± 2.66 ^bc^
Triglyceride (mg/dL)	98.25 ± 4.87	117.75 ± 5.89 ^a^	98.25 ± 3.84 ^c^	108.5 ± 12.87	100.25 ± 10.49	89.5 ± 5.42 ^c^
HDL (mg/dL)	67.25 ± 3.38	89.25 ± 3.28 ^b^	88 ± 1.41 ^b^	83.5 ± 4.41 ^a^	95.25 ± 2.69 ^b^	85.5 ± 1.19 ^b^
LDL (mg/dL)	8.75 ± 0.25	11.5 ± 0.5 ^b^	9.75 ± 0.25 ^ac^	11 ± 0.82 ^a^	12.75 ± 0.25 ^b^	10.25 ± 0.48 ^a^
Atherodenic index ^7^	1.28 ± 0.03	1.73 ± 0.1 ^b^	1.58 ± 0.05 ^a^	1.59 ± 0.04 ^b^	1.48 ± 0.04 ^a^	1.57 ± 0.05 ^a^

^1^ NFD: normal-fat diet. ^2^ HFD: high-fat diet. ^3^ Gar: Garcinia cambogia 100 mg/kg/day. ^4^ 25: BSE 25 mg/kg/day. ^5^ 50: BSE 50 mg/kg/day. ^6^ 100: BSE 100 mg/kg/day. ^7^ Atherogenic index: total cholesterol/HDL cholesterol. The statistical analysis was carried out by the use of a *t*-test. ^a^ *p* < 0.05 and ^b^ *p* < 0.01 compared with the NFD group. ^c^ *p* < 0.05 and ^d^ *p* < 0.01 compared with the HFD group. Values are the mean ± SD of six mice per group.

**Table 3:** Gradient of the mobile phase.

Time (min)	Mobile Phase A (%) ^1^	Mobile Phase B (%) ^2^
0	80	20
35	60	40
40	5	95
45	5	95
46	80	20
50	80	20

^1^ Mobile phase A, MeOH. ^2^ Mobile phase B, water (with 0.1% trifluoroacetic acid).

**Table 4:** Composition of the experimental diets.

Ingredient (g/kg)	Normal-Fat Diet	High-Fat Diet
Casein	200	265.0
L-cysteine	3	4
Corn starch	150	-
Maltodextrin	-	160
Sucrose	500	90
Cellulose	50	65.5
Soybean oil	50	30
Lard	-	310
Mineral mixture	35	48
Vitamin mixture	10	21
Choline bitartrate	2	3
Energy (kcal/g)	4	5.1
Blue food color	-	0.1
Protein (% kcal)	20	18.3
Carbohydrate (% kcal)	64	21.4
Fat (% kcal)	16	60.3

## Data Availability

The data presented in this study are available on request from the corresponding author.

## References

[1] Chooi Y.C., Ding C., Magkos F. (2019). The epidemiology of obesity. Metabolism.

[2] Landsberg L., Aronne L.J., Beilin L.J., Burke V., Igel L.I., Lloyd-Jones D., Sowers J. (2013). Obesity-related hypertension: Pathogenesis, cardiovascular risk, and treatment: A position paper of The Obesity Society and the American Society of Hypertension. J. Clin. Hypertens..

[3] Nam S.Y. (2017). Obesity-related digestive diseases and their pathophysiology. Gut Liver.

[4] Boles A., Kandimalla R., Reddy P.H. (2017). Dynamics of diabetes and obesity: Epidemiological perspective. Biochim. Biophys. Acta Mol. Basis Dis..

[5] Gallagher E.J., LeRoith D. (2015). Obesity and diabetes: The increased risk of cancer and cancer-related mortality. Physiol. Rev..

[6] De Pergola G., Silvestris F. (2013). Obesity as a major risk factor for cancer. J. Obes..

[7] Fallone F., Deudon R., Muller C., Vaysse C. (2018). Breast cancer, obesity and adipose tissue: A high-risk combination. Med. Sci..

[8] Tomiyama A.J. (2019). Stress and obesity. Ann. Rev. Psychol..

[9] Fock K.M., Khoo J. (2013). Diet and exercise in management of obesity and overweight. J. Gastroenterol. Hepatol..

[10] Saris W.H.M., Foster G.D. (2006). Simple carbohydrates and obesity: Fact, fiction and future. Int. J. Obes..

[11] Tappy L., Le K.A. (2010). Metabolic effects of fructose and the worldwide increase in obesity. Physiol. Rev..

[12] Christou G.A., Katsiki N., Blundell J., Fruhbeck G., Kiortsis D.N. (2019). Semaglutide as a promising antiobesity drug. Obes. Rev..

[13] Dong Z., Xu L., Liu H., Lv Y., Zheng Q., Li L. (2017). Comparative efficacy of five long-term weight loss drugs: Quantitative information for medication guidelines. Obes. Rev..

[14] Bessesen D.H., Van Gaal L.F. (2018). Progress and challenges in anti-obesity pharmacotherapy. Lancet Diabetes Endocrinol..

[15] Krentz A.J., Fujioka K., Hompesch M. (2016). Evolution of pharmacological obesity treatments: Focus on adverse side-effect profiles. Diabetes Obes. Metab..

[16] Bersoux S., Byun T.H., Chaliki S.S., Poole K.G. (2017). Pharmacotherapy for obesity: What you need to know. Cleve. Clin. J. Med..

[17] Costa A.G.V., Garcia-Diaz D.F., Jimenez P., Silva P.I. (2013). Bioactive compounds and health benefits of exotic tropical red–black berries. J. Funct. Foods.

[18] Kang J.G., Park C.Y. (2012). Anti-obesity drugs: A review about their effects and safety. Diabetes Metab. J..

[19] Mayer M.A., Hocht C., Puyó A., Taira C.A. (2009). Recent advances in obesity pharmacotherapy. Curr. Clin. Pharmacol..

[20] Kawk H.W., Nam G.H., Kim M.J., Kim S.Y., Kim G.N., Kim Y.M. (2020). Anti-obesity effect of an ethanol extract of cheongchunchal in vitro and in vivo. Nutrients.

[21] Im J.Y., Ki H.H., Xin M., Kwon S.U., Kim Y.H., Kim D.K., Hong S.P., Jin J.S., Lee Y.M. (2015). Anti-obesity effect of *Triticum aestivum* sprout extract in high-fat-diet-induced obese mice. Biosci. Biotechnol. Biochem..

[22] Kang N.E., Ha A.W., Woo H.W., Kim W.K. (2014). Peanut sprouts extract (*Arachis hypogaea* L.) has anti-obesity effects by controlling the protein expressions of PPARgamma and adiponectin of adipose tissue in rats fed high-fat diet. Nutr. Res. Pract..

[23] Marton M., Mandoki Z., Csapo-Kiss Z., Csapo J. (2010). The role of sprouts in human nutrition. A review. Acta Univ. Sapientiae.

[24] Lee Y.H., Kim J.H., Kim S.H., Oh J.Y., Seo W.D., Kim K.M., Jung J.C., Jung Y.S. (2016). Barley sprouts extract attenuates alcoholic fatty liver injury in mice by reducing inflammatory response. Nutrients.

[25] Byun A.R., Chun H., Lee J., Lee S.W., Lee H.S., Shim K.W. (2015). Effects of a dietary supplement with barley sprout extract on blood cholesterol metabolism. Evid.-Based Complement. Alternat. Med..

[26] Seo W.D., Yuk H.J., Curtis-Long M.J., Jang K.C., Lee J.H., Han S.I., Kang H.W., Nam M.H., Lee S.J., Lee J.H. (2013). Effect of the growth stage and cultivar on policosanol profiles of barley sprouts and their adenosine 5′-monophosphate-activated protein kinase activation. J. Agric. Food Chem..

[27] Lee J.H., Jia Y., Thach T.T., Han Y., Kim B., Wu C., Kim Y., Seo W.D., Lee S.J. (2017). Hexacosanol reduces plasma and hepatic cholesterol by activation of AMP-activated protein kinase and suppression of sterol regulatory element-binding protein-2 in HepG2 and C57BL/6J mice. Nutr. Res..

[28] Poti F., Santi D., Spaggiari G., Zimetti F., Zanotti I. (2019). Polyphenol health effects on cardiovascular and neurodegenerative disorders: A review and meta-analysis. Int. J. Mol. Sci..

[29] Arranz S., Chiva-Blanch G., Valderas-Martinez P., Medina-Remon A., Lamuela-Raventos R.M., Estruch R. (2012). Wine, beer, alcohol and polyphenols on cardiovascular disease and cancer. Nutrients.

[30] Seo K.H., Park M.J., Ra J.E., Han S.I., Nam M.H., Kim J.H., Lee J.H., Seo W.D. (2014). Saponarin from barley sprouts inhibits NF-kappaB and MAPK on LPS-induced RAW 264.7 cells. Food Funct..

[31] Vitcheva V., Simeonova R., Krasteva I., Yotova M., Nikolov S., Mitcheva M. (2011). Hepatoprotective effects of saponarin, isolated from Gypsophila trichotoma Wend. on cocaine-induced oxidative stress in rats. Redox Rep..

[32] Simeonova R., Vitcheva V., Krasteva I., Zdraveva P., Konstantinov S., Ionkova I. (2016). Antidiabetic and antioxidant effects of saponarin from Gypsophila trichotoma on streptozotocin-induced diabetic normotensive and hypertensive rats. Phytomedicine.

[33] Simeonova R., Vitcheva V., Kondeva-Burdina M., Krasteva I., Manov V., Mitcheva M. (2013). Hepatoprotective and antioxidant effects of saponarin, isolated from Gypsophila trichotoma Wend. on paracetamol-induced liver damage in rats. Biomed. Res. Int..

[34] Basile A., Giordano S., López-Sáez J.A., Cobianchi R.C. (1999). Antibacterial activity of pure flavonoids isolated from mosses. Phytochemistry.

[35] Kim J.S., Jeong E., Jo S.M., Park J., Kim J.Y. (2020). Comparative study of the effects of light controlled germination conditions on saponarin content in barley sprouts and lipid accumulation suppression in HepG2 hepatocyte and 3T3-L1 adipocyte cells using barley sprout extracts. Molecules.

[36] Engin A. (2017). Fat Cell and Fatty Acid Turnover in Obesity. Adv. Exp. Med. Biol..

[37] Ali A.T., Hochfeld W.E., Myburgh R., Pepper M.S. (2013). Adipocyte and adipogenesis. Eur. J. Cell Biol..

[38] Cristancho A.G., Lazar M.A. (2011). Forming functional fat: A growing understanding of adipocyte differentiation. Nat. Rev. Mol. Cell Biol..

[39] Jeon S.M. (2016). Regulation and function of AMPK in physiology and diseases. Exp. Mol. Med..

[40] Smith B.K., Marcinko K., Desjardins E.M., Lally J.S., Ford R.J., Steinberg G.R. (2016). Treatment of nonalcoholic fatty liver disease: Role of AMPK. Am. J. Physiol. Endocrinol. Metab..

[41] Tokarz V.L., MacDonald P.E., Klip A. (2018). The cell biology of systemic insulin function. J. Cell Biol..

[42] Carpene C., Les F., Casedas G., Peiro C., Fontaine J., Chaplin A., Mercader J., Lopez V. (2019). Resveratrol anti-obesity effects: Rapid inhibition of adipocyte glucose utilization. Antioxidants.

[43] Luo L., Liu M. (2016). Adipose tissue in control of metabolism. J. Endocrinol..

[44] Ashida H., Furuyashiki T., Nagayasu H., Bessho H., Sakakibara H., Hashimoto T., Kanazawa K. (2004). Anti-obesity actions of green tea: Possible involvements in modulation of the glucose uptake system and suppression of the adipogenesis-related transcription factors. Biofactors.

[45] Duncan R.E., Ahmadian M., Jaworski K., Sarkadi-Nagy E., Sul H.S. (2007). Regulation of lipolysis in adipocytes. Ann. Rev. Nutr..

[46] Han J.H., Jang K.W., Park M.H., Myung C.S. (2021). Garcinia cambogia suppresses adipogenesis in 3T3-L1 cells by inhibiting p90RSK and Stat3 activation during mitotic clonal expansion. J. Cell Physiol..

[47] Corey R., Werner K.T., Singer A., Moss A., Smith M., Noelting J., Rakela J. (2016). Acute liver failure associated with Garcinia cambogia use. Ann. Hepatol..

[48] Ferreira V., Mathieu A., Soucy G., Giard J.M., Erard-Poinsot D. (2020). Acute severe liver injury related to long-term garcinia cambogia intake. ACG Case Rep. J..

[49] Kothadia J.P., Kaminski M., Samant H., Olivera-Martinez M. (2018). Hepatotoxicity associated with use of the weight loss supplement garcinia cambogia: A case report and review of the literature. Case Rep. Hepatol..

[50] Lemieux I., Lamarche B., Couillard C., Pascot A., Cantin B., Bergeron J., Dagenais G.R., Després J.P. (2001). Total cholesterol/HDL cholesterol ratio vs LDL cholesterol/HDL cholesterol ratio as indices of ischemic heart disease risk in men: The Quebec Cardiovascular Study. Arch. Intern. Med..

[51] Derosa G., Maffioli P. (2012). Anti-obesity drugs: A review about their effects and their safety. Expert Opin. Drug Saf..

[52] Son J.W., Kim S. (2020). Comprehensive review of current and upcoming anti-obesity drugs. Diabetes Metab. J..

[53] Park B., Lee S., Lee B., Kim I., Baek N., Lee T.H., Lee S.Y., Son M., Park H. (2016). New ethanol extraction improves the anti-obesity effects of black tea. Arch. Pharm. Res..

[54] Onyebuchi C., Kavaz D. (2020). Effect of extraction temperature and solvent type on the bioactive potential of *Ocimum gratissimum* L. extracts. Sci. Rep..

[55] Lee Y.M., Kim D.S. (2020). The extraction solvent influences the anti-inflammatory effects of jakyakgamcho-tang in lipopolysaccharide-stimulated macrophages and mice with gouty arthritis. Int J. Mol. Sci..

[56] Kolb H., Kempf K., Rohling M., Martin S. (2020). Insulin: Too much of a good thing is bad. BMC Med..

[57] Ludwig D.S., Ebbeling C.B. (2018). The carbohydrate-insulin model of obesity: Beyond “calories in, calories out”. JAMA Intern. Med..

[58] Van Dam R.M., Seidell J.C. (2007). Carbohydrate intake and obesity. Eur. J. Clin. Nutr..

[59] Mukherjee S., Thakur G., Kumar B.D., Mitra A., Chakraborty C. (2009). Long-term effects of a carbohydrate-rich diet on fasting blood sugar, lipid profile, and serum insulin values in rural Bengalis. J. Diabetes.

[60] Borer K.T. (2019). Understanding human physiological limitations and societal pressures in favor of overeating helps to avoid obesity. Nutrients.

[61] Miller W.C., Lindeman A.K., Wallace J., Niederpruem M. (1990). Diet composition, energy intake, and exercise in relation to body fat in men and women. Am. J. Clin. Nutr..

[62] Koh Y.M., Jang S.W., Ahn T.W. (2019). Anti-obesity effect of Yangkyuksanwha-tang in high-fat diet-induced obese mice. BMC Complement. Altern. Med..

[63] Pandeya P.R., Lamichhane R., Lee K.H., Lamichhane G., Kim S.G., Jung H.J. (2021). Efficacy of a novel herbal formulation (F2) on the management of obesity: In vitro and in vivo study. Evid.-Based Complement. Alternat. Med..

[64] Lee C.L., Wen J.Y., Hsu Y.W., Pan T.M. (2013). Monascus-fermented yellow pigments monascin and ankaflavin showed antiobesity effect via the suppression of differentiation and lipogenesis in obese rats fed a high-fat diet. J. Agric. Food Chem..

[65] Achari A.E., Jain S.K. (2017). Adiponectin, a therapeutic target for obesity, diabetes, and endothelial dysfunction. Int. J. Mol. Sci..

[66] Schwartz G.J., Azzara A.V., Heaner M.K. (2013). Roles for central leptin receptors in the control of meal size. Appetite.

[67] Krishnan S., Tryon R.R., Horn W.F., Welch L., Keim N.L. (2016). Estradiol, SHBG and leptin interplay with food craving and intake across the menstrual cycle. Physiol. Behav..

[68] Pan H., Guo J., Su Z. (2014). Advances in understanding the interrelations between leptin resistance and obesity. Physiol. Behav..

[69] Benetolo P.O., Fernandes M.I.M., Ciampo I., Elias-Junior J., Sawamura R. (2019). Evaluation of nonalcoholic fatty liver disease using magnetic resonance in obese children and adolescents. J. Pediatr..

